# A mathematical model for the transmission of co-infection with COVID-19 and kidney disease

**DOI:** 10.1038/s41598-024-56399-2

**Published:** 2024-03-07

**Authors:** Md. Abdul Hye, Md. Haider Ali Biswas, Mohammed Forhad Uddin, Md. M. Rahman

**Affiliations:** 1 Department of Mathematics and Statistics, Bangladesh University of Business and Technology (BUBT), Dhaka, 1216 Bangladesh; 2 Mathematics Discipline, Khulna University, Khulna, 9208 Bangladesh; 3 Department of Mathematics, Bangladesh University of Engineering and Technology (BUET), Dhaka, 1000 Bangladesh; 4 Department of Mathematics, Faculty of Science, Islamic University, Kushtia, 7003 Bangladesh; 5 School of Engineering, Design and Built Environment, Western Sydney University, Penrith, NSW 2751 Australia

**Keywords:** Co-infection, Kidney disease, COVID-19, Numerical solution, Sensitivity analysis, Parameter estimation, Computational biology and bioinformatics, Diseases, Mathematics and computing

## Abstract

The world suffers from the acute respiratory syndrome COVID-19 pandemic, which will be scary if other co-existing illnesses exacerbate it. The co-occurrence of the COVID-19 virus with kidney disease has not been available in the literature. So, further research needs to be conducted to reveal the transmission dynamics of COVID-19 and kidney disease. This study aims to create mathematical models to understand how COVID-19 interacts with kidney diseases in specific populations. Therefore, the initial step was to formulate a deterministic Susceptible-Infected-Recovered (SIR) mathematical model to depict the co-infection dynamics of COVID-19 and kidney disease. A mathematical model with seven compartments has been developed using nonlinear ordinary differential equations. This model incorporates the invariant region, disease-free and endemic equilibrium, along with the positivity solution. The basic reproduction number, calculated via the next-generation matrix, allows us to assess the stability of the equilibrium. Sensitivity analysis is also utilised to understand the influence of each parameter on disease spread or containment. The results show that a surge in COVID-19 infection rates and the existence of kidney disease significantly enhances the co-infection risks*.* Numerical simulations further clarify the potential outcomes of treating COVID-19 alone, kidney disease alone, and co-infected cases. The study of the potential model can be utilised to maximise the benefits of simulation to minimise the global health complexity of COVID-19 and kidney disease.

## Introduction

The latest coronavirus disease 2019 (COVID-19) is a continuous, highly common infectious caused by the Severe Respiratory Syndrome Coronavirus 2 (SARS-CoV-2) that originated in China in 2019^1–3^ has indeed spread to over 300 million people and has occurred about 6 million annual deaths. COVID-19 mainly spreads through the inhalation of infectious respiratory droplets. Additionally, it can be spread by coming into contact with possibly contaminated body parts or surfaces and then consuming the virus (Kutter et al., 2018; Andersen et al., 2020), causing chills, coughing, exhaustion, body pains, headaches, loss of taste or smell, sore throat, and shortness of breath are some of the clinical signs of COVID-19. These manifestations can lead to illnesses of varying severity, potentially resulting in death^4^. Age and the presence of underlying health vulnerabilities, such as cancer, kidney disease, lung ailments, neurological disorders, diabetes, and heart conditions, reduce the body's ability to fight off the COVID-19 virus. These factors increase the risk of hospitalisation and death from COVID-19 as well as the vulnerability of clinical signs^5–8^. In a recent study examining the transmission dynamic of COVID-19 with dengue co-infection, mathematical modelling was employed to gain insight into the combined effect of the two diseases^9^. Conventional strategies to control COVID-19 focus on minimising contact. This includes isolating the sick, using protective measures like gloves and face masks, and practising quarantine.^10–12^. However, various vaccines have recently been formulated and distributed to curb the spread of COVID-19 further. Most countries are experiencing the fourth wave of the virus despite persistent efforts to contain it. Challenges such as limited vaccine availability, hesitancy towards vaccination, questions about vaccine effectiveness, waning vaccine immunity, non-adherence to public health guidelines, and viral mutations have impeded the success of vaccination and other preventive measures.

COVID-19 triggers both lower and upper respiratory tract infections, which can result in pneumonia. Additionally, the virus can affect various other tissues and organs, including the kidneys^13^. As per Hu, et al.^14^, COVID-19 can lead to multi-organ failure, heightening the mortality risk, particularly in patients with chronic ailments. A significant number of patients in intensive care units (ICU) are confirmed cases with underlying co-morbidities. Many studies have recently been conducted to investigate the impact of COVID-19 on kidney disease and patient outcomes^14^. In a prospective study, Cheng, et al.^15^ discovered that 2% of confirmed cases have chronic kidney disease. Due to their immunosuppression, patients who have had kidney transplants are also affected by SARS-CoV-2^13^. One common factor that affects the severity of the disease and the risk of death in patients is acute kidney injury (AKI)^16^.

Saha, et al.^17^ report that their model exhibits transcritical, backward, and forward bifurcations with hysteresis. They validated this model with COVID-19 data from Hong Kong (December 19 2021 to April 3 2022), estimated essential parameters, identified sensitive parameters, calculated R(t) for the same period, and analysed an optimal control problem with vaccination to determine the best strategies for reducing the disease's impact on the population and minimising associated costs. Biswas, et al.^18^ analysed the spread of COVID-19 in high-density India using a compartmental model, focusing on parameter estimation, sensitivity analysis, and effective prevention strategies. Asamoah, et al.^19^ explained a mathematical model for controlling gonorrhea transmission, incorporating techniques like education, condom use, vaccination, and treatment, and demonstrated through simulations that these measures effectively reduce infection rates. Asamoah, et al.^20^ focused on mathematical models, both integer and fractional order, to effectively analyse the dynamics of Q fever transmission in livestock involving ticks, with the Atangana-Baleanu operator showing better performance in capturing susceptibilities and reducing infections. Asamoah, et al.^21^ presented research on the global stability and cost-effectiveness of COVID-19 management strategies, particularly considering environmental impacts, and used data from Ghana for its analysis. Asamoah, et al.^22^ conducted an in-depth analysis of optimal control strategies and their cost-effectiveness in managing COVID-19. Furthermore, Asamoah, et al.^23^ were involved in a detailed sensitivity assessment and economic evaluation of a novel compartmental model for COVID-19, incorporating various control interventions.

A vaccination model for COVID-19 includes environmental transmission, focusing on the model's stability based on Pfizer vaccination data in Nigeria and observing the effects of varying fractional-order values on model^24^. The study explores the impact of COVID-19 and dengue vaccinations on Zika transmission through a vaccination model, highlighting the positive influence of increased vaccination efforts on Zika dynamics and the co-spread of these infections, based on data from Amazonas, Brazil^25^. The study enhanced preventive measures against incident co-infection of SARS-CoV-2 and HBV, which can significantly control their co-circulation, as concluded from a co-dynamical model and numerical assessments focusing on various intervention strategies^26^.

Despite the widespread impact of COVID-19, there's been limited research on its co-infection with kidney disease. Therefore, the co-infection of the COVID-19 virus with kidney disease remains notably unexplored. This study delves into the complex interplay between COVID-19 and kidney disease, addressing a significant gap in current medical research. The paper is structured as follows: firstly, we present a detailed overview of COVID-19's global impact, its transmission methods, clinical symptoms, and the increased risks associated with co-morbidities, specifically kidney disease. Secondly, we introduce a mathematical model elucidating the transmission dynamics of COVID-19 when co-infected with kidney disease. The approach involves constructing a Susceptible-Infected-Recovered (SIR) model encapsulated within a seven-compartment framework based on nonlinear ordinary differential equations. Thirdly, we analyse the disease's dynamics, including assessing equilibrium states and their stability and conducting a sensitivity analysis to understand the impact of various parameters on the disease's spread or containment. The culmination of our study presents insightful findings on the risks and management strategies of these co-infections, thereby contributing significantly to the broader understanding and handling of such complex medical scenarios.

## Model formulation

**Figure 1:**
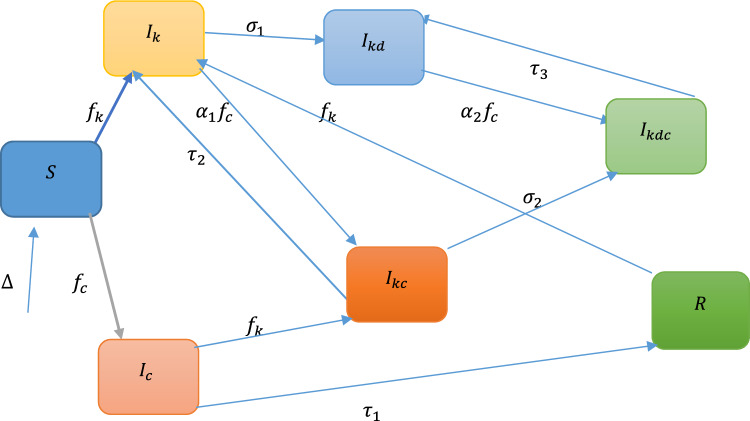
Flow chart for the transmission dynamics of the co-infection of COVID-19 with kidney disease.

We consider a deterministic seven-compartmental human population (Fig. 1). The total population is divided into seven sub-classes, which are susceptible population (S), infectious individuals with COVID-19 $({I}_{c})$, infected by the primary stage of the kidney $({I}_{k}),$ infected by end-stage kidney disease $({I}_{kd})$, co-infected with COVID-19 and primary stage of kidney disease $\left({I}_{kc}\right)$,co-infected with COVID-19 and end-stage kidney disease $\left({I}_{kdc}\right)$, individuals who have recovered from COVID-19 $(R)$. We assume that the rate of increase in the susceptible population stems from a recruitment rate represented by $\Delta$, while there's a natural mortality rate $\mu$ present across all classes. In the total susceptible population, individuals can get kidney disease with a contact rate of ${\phi }_{2}$ from a kidney disease only infected or co-infected person with the force of infection of kidney disease, ${f}_{k}=\frac{{\phi }_{2}[{I}_{k}+\theta \left({I}_{kd}+{I}_{kc}+{I}_{kdc}\right)]}{N}$, and join ${I}_{k}$ state variable. Similarly, individuals can get COVID-19 with a contact rate of ${\phi }_{1}$ from a COVID-19-only infected or co-infected person with the force of infection of COVID-19 ${f}_{c}=\frac{{\phi }_{1}[{I}_{c}+\gamma \left({I}_{kdc}+{I}_{kc}\right)]}{N}$, and join ${I}_{c}$ state variable. Kidney disease only infected individuals can also get an additional COVID-19 infection with the force of infection ${f}_{c}$ and join the co-infected compartment $({I}_{kc})$.The co-infected compartment increases because individuals that come from COVID-19 only infected compartment when kidneys infect them with ${f}_{k}$ the force of infection. In this context, θ is the parameter adjusting for the enhanced transmission of kidney disease among co-infected individuals and those in the end-stage of the disease, and γ denotes the parameter accounting for the amplified transmissibility of COVID-19 in co-infected persons. Parameters ${\sigma }_{1}$,${\sigma }_{2}$ are represented progression rates to fully increased kidney disease by compartments ${I}_{k}$ and ${I}_{kc}$, respectively. The parameters ${\tau }_{1},{\tau }_{2}$ and ${\tau }_{3}$ indicate recovery rates from COVID-19 for individuals in compartments $({I}_{c} )$_,_ co-infected with COVID-19 and primary stage of kidney disease $\left({I}_{kc}\right)$, co-infected with COVID-19 and end-stage kidney disease $\left({I}_{kdc}\right)$ respectively and ${\alpha }_{1}$,${\alpha }_{2}$ parameters denote adjustments for the susceptibility of individuals with kidney disease to contracting a COVID-19 infection.

1 $$\begin{gathered} \frac{dS}{{dt}} = \Delta - \frac{{\phi_{2} \left[ {I_{k} + \theta \left( {I_{kd} + I_{kc} + I_{kdc} } \right)} \right]}}{N}S - \frac{{\phi_{1} \left[ {I_{c} + \gamma \left( {I_{kdc} + I_{kc} } \right)} \right]}}{N}S - \mu S \hfill \\ \frac{{dI_{k} }}{dt} = \frac{{\phi_{2} \left[ {I_{k} + \theta \left( {I_{kd} + I_{kc} + I_{kdc} } \right)} \right]}}{N}S - \sigma_{1} I_{k} - \alpha_{1} \frac{{\phi_{1} \left[ {I_{c} + \gamma \left( {I_{kdc} + I_{kc} } \right)} \right]}}{N}I_{k} + \frac{{\phi_{2} \left[ {I_{k} + \theta \left( {I_{kd} + I_{kc} + I_{kdc} } \right)} \right]}}{N}R + \tau_{2} I_{kc} - \mu I_{k} \hfill \\ \frac{{ dI_{c} }}{dt} = \frac{{\phi_{1} \left[ {I_{c} + \gamma \left( {I_{kdc} + I_{kc} } \right)} \right]}}{N}S - \frac{{\phi_{2} \left[ {I_{k} + \theta \left( {I_{kd} + I_{kc} + I_{kdc} } \right)} \right]}}{N}I_{c} - \tau_{1} I_{c} - \mu I_{c} \hfill \\ \frac{{ dI_{kc} }}{dt} = \alpha_{1} \frac{{\phi_{1} \left[ {I_{c} + \gamma \left( {I_{kdc} + I_{kc} } \right)} \right]}}{N}I_{k} + \frac{{\phi_{2} \left[ {I_{k} + \theta \left( {I_{kd} + I_{kc} + I_{kdc} } \right)} \right]}}{N}I_{c} - \sigma_{2} I_{kc} - \tau_{2} I_{kc} - \mu I_{kc} \hfill \\ \frac{{dI_{kd} }}{dt} = \sigma_{1} I_{k} + \tau_{3} I_{kdc} - \alpha_{2} \frac{{\phi_{1} \left[ {I_{c} + \gamma \left( {I_{kdc} + I_{kc} } \right)} \right]}}{N} I_{kd} - \mu I_{kd} \hfill \\ \frac{{dI_{kdc} }}{dt} = \sigma_{2} I_{kc} + \alpha_{2} \frac{{\phi_{1} \left[ {I_{c} + \gamma \left( {I_{kdc} + I_{kc} } \right)} \right]}}{N} I_{kd} - \tau_{3} I_{kdc} - \mu I_{kdc} \hfill \\ \frac{dR}{{dt}} = \tau_{1} I_{c} - \frac{{\phi_{2} \left[ {I_{k} + \theta \left( {I_{kd} + I_{kc} + I_{kdc} } \right)} \right]}}{N}R - \mu R \hfill \\ \end{gathered}$$

## Analytical analysis

We studied how COVID-19 and kidney disease impact each other by examining them separately first. After understanding each individually, they’re combined to see the overall effect. The goal is to ensure the combined results are accurate and logical.

COVID-19-only model: when we exclude kidney disease infections, we can formulate a COVID-19-specific sub-model from the full disease model; we get ${I}_{k}=0,{I}_{kc}=0,{I}_{kd}=0,{I}_{kdc}=0$

2 $$\begin{gathered} \frac{dS}{{dt}} = \Delta - \frac{{\phi_{1} I_{c} }}{N}S - \mu S \hfill \\ \frac{{dI_{c} }}{dt} = \frac{{\phi_{1} I_{c} }}{N}S - \tau_{1} I_{c} - \mu I_{c} \hfill \\ \frac{dR}{{dt}} = \tau_{1} I_{c} - \mu R \hfill \\ \end{gathered}$$

### Theorem 1

All the populations of the system with positive initial conditions are nonnegative.

Assume ${\text{S}}\left(0\right)>0,{{\text{I}}}_{{\text{C}}}(0)>0,{\text{R}}(0)>0$ are positive for time ${\text{t}}> 0$ and for all nonnegative parameters.

From the initial condition, all the state variables are nonnegative at the initial time; then, $\mathrm{t }> 0$

To show the solutions of the model, as it is positive, first, we take $\frac{{\text{dS}}}{{\text{dt}}}$ from equation

$$\frac{{\text{dS}}}{{\text{dt}}}=\Delta -\frac{{\upphi }_{1}{{\text{I}}}_{{\text{c}}}}{{\text{N}}}{\text{S}}-\mathrm{\mu S}$$

$$\frac{{\text{ds}}}{{\text{dt}}}=\Delta -\left(\frac{{\upphi }_{1}{{\text{I}}}_{{\text{c}}}}{{\text{N}}}+\upmu \right){\text{s}}$$

$${\text{S}}\left({\text{t}}\right)={\text{S}}(0){\text{exp}}\left(-\underset{0}{\overset{{\text{t}}}{\int }}\left\{\frac{{\upphi }_{1}{{\text{I}}}_{{\text{c}}}}{{\text{N}}}+\upmu \right\}{\text{du}}\right)+\underset{0}{\overset{{\text{t}}}{\int }}\Delta \mathrm{ exp}(\underset{0}{\overset{{\text{x}}}{\int }}\left\{\frac{{\upphi }_{1}{{\text{I}}}_{{\text{c}}}}{{\text{N}}}+\upmu \right\}{\text{du}})\mathrm{dx }\times {\text{exp}}\left(-\underset{0}{\overset{{\text{t}}}{\int }}\left\{\frac{{\upphi }_{1}{{\text{I}}}_{{\text{c}}}}{{\text{N}}}+\upmu \right\}{\text{du}}\right)>0$$

Accordingly, all the variables are nonnegative in $[0,{\text{t}}]$, so ${\text{S}}\left(0\right)>0,$ similarly we can show ${{\text{I}}}_{{\text{C}}}(0)>0,{\text{R}}(0)>0$.

### Theorem 2

The dynamical system represented by the COVID-19 submodel remains positively invariant within the closed invariant set defined by ${\rm Z}_{c} = \left\{\left( S,{I}_{c},R\right)\epsilon {R}^{3}+ : N\le \frac{\Delta }{\mu }\right\}$

An invariant region is identified to demonstrate that the solution remains within certain bounds. This invariant region provides a constraint ensuring that the solution does not exceed these limits; we have

$$N=S+{I}_{C}+R$$

$$\frac{dN}{dt}=\frac{dS}{dt}+\frac{{dI}_{C}}{dt}+\frac{dR}{dt}$$

$$\frac{dN}{dt}=\Delta -\frac{{\phi }_{1}{I}_{c}}{N}S-\mu S+\frac{{\phi }_{1}{I}_{c}}{N}S-{\tau }_{1}{I}_{c}-\mu {I}_{c}+{\tau }_{1}{I}_{c}-\mu R$$

$$\frac{dN}{dt}=\Delta -\left(S+{I}_{c}+R\right)\mu$$

$$\frac{dN}{dt}=\Delta -N\mu$$

$$N\left(t\right)=N\left(0\right){e}^{-\mu t}+\frac{\Delta }{\mu }(1-{e}^{-\mu t})$$

As,$t\to \infty$, we get $0\le N\le \frac{\Delta }{\mu }$, the theory of differential equation^27^ in the region.

${\rm Z}_{c} = \{\left( S,{I}_{c},R\right)\epsilon {R}^{3}+ : N\le \frac{\Delta }{\mu } \}$, For the autonomous system representing the COVID-19-only model, given by (2), any solution that starts in ${Z}_{c}$ will stay within ${Z}_{c}$ for all $t\ge 0.$ Based on Cheng et al., this means that ${Z}_{c}$ acts as a stable and attractive region. Therefore, according to Naicker et al., the dynamics of model (2) are both mathematically sound and relevant to epidemiology, and it is appropriate to study its tabiliz within ${Z}_{c}.$

**Stability analysis of equilibrium states:** In the only COVID-19 sub-model, the equilibrium state is reached when the following conditions are met

$$\frac{dS}{dt}=\frac{{dI}_{c}}{dt}=\frac{dR}{dt}=0$$

For the isolated COVID-19 model represented by the system (2), the state without any active disease (termed the disease-free equilibrium or DFE) is derived by setting each component of the system (2) to zero. At this DFE, neither infections nor recoveries are present.

Therefore, for the stand-alone COVID-19 model (2), the DFE is described ${\Omega }_{c}=\left(S,{I}_{C},R\right)=(\frac{\Delta }{\mu },\mathrm{0,0})$

The sub-model’s basic reproduction number is the average number of secondary infections caused by a single COVID-19-infected person in a totally susceptible population. The system (2) calculates it using the next-generation matrix.

3 $${R}_{oc}=\frac{{\phi }_{1}}{({\tau }_{1}+\mu )}$$

The basic reproduction number, ${R}_{0c}$, represents the average number of people one infected individual is expected to infect over their entire infectious period within a completely susceptible population.

### Theorem 3

For the kidney disease sub-model, the point of equilibrium without the disease is represented as ${\Omega }_{0c}$, remains stable as long as the basic reproduction number ${R}_{oc}$ is less than 1.

The Jacobian matrix is tabiliz to ascertain the equilibrium points’ local stability. For sub-model (2), the Jacobian matrix is formulated as $$J=\left(\begin{array}{c}\frac{\partial {f}_{1}}{\partial S} \frac{\partial {f}_{1}}{\partial {I}_{C}} \frac{\partial {f}_{1}}{R}\\ \frac{\partial {f}_{2}}{\partial S} \frac{\partial {f}_{2}}{\partial {I}_{C}} \frac{\partial {f}_{2}}{R} \\ \frac{\partial {f}_{3}}{\partial S} \frac{\partial {f}_{3}}{\partial {I}_{C}} \frac{\partial {f}_{3}}{R}\end{array}\right)$$

$$J=\left(\begin{array}{c}-\frac{{\varnothing }_{1}{I}_{c}}{N}-\mu \frac{{\varnothing }_{1}S}{N} \,\,\,\,\,\,\,0\\ \frac{{\varnothing }_{1}{I}_{c}}{N} -{\tau }_{1}-\mu \,\,\,\,\,\,\,\,\,\,0 \\ 0 \,\,\,\,\,\,\,\,\,\,{\tau }_{1} -\mu \end{array}\right)$$

The Jacobian matrix for the sub-model, when evaluated at the disease-free equilibrium point ${\Omega }_{0c}$, is expressed as

$$J({\Omega }_{0c})=\left(\begin{array}{c}-\mu \,\,\,\,\,\,\,\,\frac{{\varnothing }_{1}\Delta }{\mu N} \,\,\,\,\,\,\,0 \\ 0 \,\,\,\,\,-\left({\tau }_{1}+\mu \right) \,\,\,\,\,\,\,0\\ 0 \,\,\,\,\,\,\,\,{\tau }_{1} -\mu \end{array}\right)$$

In this context, one of the eigenvalues for ${\Omega }_{0c}$ is $\lambda =-\mu$. The other eigenvalues can be conveniently derived from the associated submatrix.

$${J}_{1}=\left(\begin{array}{cc}-\left({\tau }_{1}+\mu \right)& 0\\ {\tau }_{1}& -\mu \end{array}\right)$$

To confirm the local stability of the disease-free equilibrium point, two conditions need to be met:

(i) The trace of ${J}_{1}$ should be less than zero. (ii) The determinant of ${J}_{1}$ should be greater than zero.

The trace is Trc $\left({J}_{1}\right)=-({\tau }_{1}+2\mu ),$ which is less than zero.

$${\text{det}}\left({J}_{1}\right)=\left({\tau }_{1}+\mu \right)\mu >0$$

As a result, the COVID-19 sub-model’s disease-free equilibrium point is asymptotically stable.

Theorem 5. The COVID-19 submodel has an isolated endemic equilibrium point if ${R}_{0c}>1$.

The endemic equilibrium point of the COVID-19 sub-model is the solution of the system of equation in (4).

$$\Delta -\left({{\text{f}}}_{{\text{c}}}+\mu \right)S=0$$

$${f}_{c}S-\left({\tau }_{1}+\mu \right){I}_{c}=0$$

$${\tau }_{1}{I}_{c}-\mu R=0$$

To solve this system of equations,

we express it in terms of

4 $${f}_{c}^{*}=\frac{{\phi }_{1}{I}_{c}^{*}}{N}$$

5 $${S}^{*}=\frac{\Delta }{{f}_{c}^{*}+\mu }, {I}_{c}^{*}=\frac{{f}_{c}^{*}S}{({\tau }_{1}+\mu )}, {R}^{*}=\frac{{\tau }_{1}{I}_{c}*}{\mu },$$

Now,

$${I}_{c}^{*}=\frac{{f}_{c}^{*}S}{({\tau }_{1}+\mu )}$$

$${I}_{c}^{*}=\frac{\Delta {f}_{c}^{*}}{({\tau }_{1}+\mu )({f}_{c}^{*}+\mu )}$$

So, using (4)

$${f}_{c}^{*}=\frac{{\phi }_{1}{I}_{c}^{*}}{N}$$

$${f}_{c}^{*}=\frac{{\phi }_{1}\mu }{\left({\tau }_{1}+\mu \right)}-\mu$$

$${f}_{c}^{*}=\mu (\frac{{\phi }_{1}}{\left({\tau }_{1}+\mu \right)}-1)$$

$${f}_{c}^{*}=\mu ({R}_{0c}-1)$$

The conclusion drawn is that the infection force ${f}_{c}^{*}$ will be positive at the endemic equilibrium point ${\Omega }_{0c}$ only when ${R}_{oc}>1$. With this, we have effectively demonstrated the related theorem.

### Theorem 5

Analysis of the Global Stability Analysis for the Endemic Equilibrium Point.

The endemic equilibrium point ${\Omega }_{c}$ undergoes a global stability analysis using the Lyapunov function method. To facilitate this analysis, we establish the

6 $$L=\frac{1}{2}((S-{S}^{*})+\left({I}_{c}-{I}_{c}^{*}\right)+{\left(R-{R}^{*}\right))}^{2}$$

The Lyapunov function L consistently maintains a positive value and only becomes zero at the endemic equilibrium point and differentiating with respect to time $t$

$$\begin{gathered} \frac{{{\text{dL}}}}{{{\text{dt}}}} = \left\{ {\left( {{\text{S}} - {\text{S}}^{*} } \right) + \left( {{\text{I}}_{{\text{c}}} - {\text{I}}_{{\text{c}}}^{*} } \right) + \left( {{\text{R}} - {\text{R}}^{*} } \right)} \right\}\left( {\frac{{{\text{dS}}}}{{{\text{dt}}}} + \frac{{{\text{dI}}_{{\text{c}}} }}{{{\text{dt}}}} + \frac{{{\text{dR}}}}{{{\text{dt}}}}} \right) \hfill \\ = \left\{ {\left( {{\text{S}} + {\text{I}}_{{\text{c}}} + {\text{R}}} \right) - \left( {{\text{S}}^{*} + {\text{I}}_{{\text{c}}}^{*} + {\text{R}}^{*} } \right)} \right\}\left( {\Delta - {\mu N}} \right) \hfill \\ = \frac{{\left( {{\mu N} - \Delta } \right)}}{{\upmu }}\left( {\Delta - {\mu N}} \right) \hfill \\ = - \frac{{\left( {\Delta - {\mu N}} \right)^{2} }}{{\upmu }} \hfill \\ \frac{{{\text{dL}}}}{{{\text{dt}}}} \le 0 \hfill \\ \end{gathered}$$

For ${R}_{oc}>1$, the endemic equilibrium point exists, leading to $\frac{dL}{dt}$ is less than zero. It seems that the function L appears as a clear-cut Lyapunov function, suggesting that the endemic equilibrium point reaches asymptotic and global stability. From a biological perspective, this signifies that COVID-19 has remained prevalent in the community over a prolonged duration.

### Analysing the sensitivity-only COVID-19 model

We conducted a sensitivity analysis of parameters within the COVID-19 sub-model. The behavior of the model in response to modest changes in a parameter’s value is known as the parameter’s sensitivity and is tabilize by the symbol ${\phi }_{1}$. It can be expressed as

$${R}_{oc}=\frac{{\phi }_{1}}{\left({\tau }_{1}+\mu \right)}$$

$${S}_{{\phi }_{1}}=\frac{\partial {R}_{0c}}{\partial {\varnothing }_{1}} \frac{{\phi }_{1}}{{R}_{0c}}=\frac{1}{\left({\tau }_{1}+\mu \right)} \frac{{\phi }_{1}}{\frac{{\phi }_{1}}{\left({\tau }_{1}+\mu \right)}}=+1$$

$${S}_{\mu }=\frac{\partial {R}_{0c}}{\partial \mu } \frac{\mu }{{R}_{0c}}= - \frac{{\phi }_{1}}{{\left({\tau }_{1}+\mu \right)}^{2}} \frac{\mu }{\frac{{\phi }_{1}}{\left({\tau }_{1}+\mu \right)}}=-\frac{\mu }{(\mu +{\tau }_{1})}$$

$${S}_{{\tau }_{1} }=\frac{\partial {R}_{0c}}{\partial {\tau }_{1}} \frac{{\tau }_{1}}{{R}_{0c}}=-\frac{{\phi }_{1}}{{\left({\tau }_{1}+\mu \right)}^{2}} \frac{{\tau }_{1}}{\frac{{\phi }_{1}}{\left({\tau }_{1}+\mu \right)}}=-\frac{{\tau }_{1}}{\left({\tau }_{1}+\mu \right)}$$

**Figure 2:**
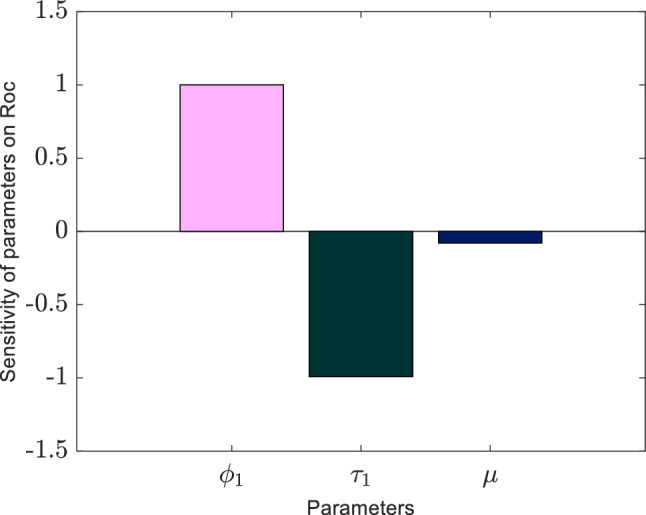
The graphical depiction of the sensitivity indices concerning the primary reproduction number $({R}_{oc})$ parameters are shown in the COVID-19 sub-model.

Table 1 displays the data for the sensitivity indices related to the sole COVID-19 sub-model. This sub-model analysis reveals that the COVID-19 contact rate is ${\phi }_{1}$, play a significant role in intensifying the virus’s spread. This trend results from an upsurge in secondary infections when these parameters increase, as highlighted by (Martcheva 2015). Conversely, parameters such as ${\tau }_{1}$ and $\mu$ have a diminishing effect, meaning an uptick in their values could reduce the infection rate. A visual depiction of the sensitivity indices for ${R}_{oc}$ is showcased in Fig. 2.

**Table 1:** Values indicated in Table 3 were used to compute the sensitivity indices for the only COVID-19 sub-model.

Parameter	Description of the parameter	Measures of sensitivity
${\tau }_{1}$	COVID-19 single-infection recovery rate	$-0.99112$
${\phi }_{1}$	Contact rate of Covid-19	+ 1
$\mu$	Human natural death rate	$-0.08$

### Kidney disease-only model

Kidney disease-only sub-model from the co-infection model, we get ${I}_{c}=0,{I}_{kc}=0,{I}_{kdc}=0,R=0$

7 $$\begin{gathered} \frac{dS}{{dt}} = \Delta - f_{k} S - \mu S \hfill \\ \frac{{dI_{k} }}{dt} = f_{k} S - \sigma_{1} I_{k} - \mu I_{k} \hfill \\ \frac{{dI_{kd} }}{dt} = \sigma_{1} I_{k} - \mu I_{kd} \hfill \\ \end{gathered}$$

#### Theorem 6

All the populations of the system with positive initial conditions are nonnegative.

Assume ${\text{S}}(0) > 0,{{\text{I}}}_{{\text{k}}}(0) >0,{{\text{I}}}_{{\text{k}}}(0) > 0$ are positive for time $\mathrm{t }>0$ and all nonnegative parameters.

From the initial condition, all the state variables are nonnegative at the initial time; then, $\mathrm{t }>0$.

To show the solutions of the model, as it is positive, first, we take $\frac{{\text{dS}}}{{\text{dt}}}$ from equation

8 $$\begin{gathered} \frac{{{\text{dS}}}}{{{\text{dt}}}} = \Delta - \frac{{\phi_{2} {\text{I}}_{{\text{k}}} }}{{\text{N}}}{\text{S}} - {\mu S} \hfill \\ \frac{{{\text{dS}}}}{{{\text{dt}}}} = \Delta - \left( {\frac{{\phi_{2} {\text{I}}_{{\text{k}}} }}{{\text{N}}} + {\upmu }} \right){\text{S}} \hfill \\ {\text{S}}\left( {\text{t}} \right) = {\text{S}}\left( 0 \right)\exp \left( { - \mathop \smallint \limits_{0}^{{\text{t}}} \left\{ {\frac{{\phi_{2} {\text{I}}_{{\text{k}}} }}{{\text{N}}} + {\upmu }} \right\}{\text{du}}} \right) + \mathop \smallint \limits_{0}^{{\text{t}}} \Delta {\text{ exp}}(\mathop \smallint \limits_{0}^{{\text{x}}} \left\{ {\frac{{\phi_{2} {\text{I}}_{{\text{k}}} }}{{\text{N}}} + {\upmu }} \right\}{\text{du}}){\text{dx }} \times \exp \left( { - \mathop \smallint \limits_{0}^{{\text{t}}} \left\{ {\frac{{\phi_{2} {\text{I}}_{{\text{k}}} }}{{\text{N}}} + {\upmu }} \right\}{\text{du}}} \right) > 0 \hfill \\ \end{gathered}$$

Hence ${\text{S}}(0)>0$, similarly we can prove ${{\text{I}}}_{{\text{k}}}(0) >0,{\mathrm{ I}}_{{\text{k}}}(0) > 0$.

#### Theorem 7

The dynamical system (7) is positively invariant in the closed invariant set.

$${\rm Z}_{k} = \{\left( S,{I}_{k},{I}_{kd}\right)\epsilon {R}^{3}+ : N\le \frac{\Delta }{\mu } \}$$

To obtain an invariant region that shows that the solution is bounded, we have

$$\begin{gathered} N = S + I_{k} + I_{kd} \hfill \\ \frac{dN}{{dt}} = \frac{dS}{{dt}} + \frac{{dI_{k} }}{dt} + \frac{{dI_{kd} }}{dt} \hfill \\ \frac{dN}{{dt}} = \Delta - f_{k} S - \mu S + f_{k} S - \sigma_{1} I_{k} - \mu I_{k} + \sigma_{1} I_{k} - \mu I_{kd} \hfill \\ \frac{dN}{{dt}} = \Delta - \left( {S + I_{k} + I_{kd} } \right)\mu \hfill \\ \frac{dN}{{dt}} = \Delta - N\mu \hfill \\ N\left( t \right) = N\left( 0 \right)e^{ - \mu t} + \frac{\Delta }{\mu }\left( {1 - e^{ - \mu t} } \right) \hfill \\ \end{gathered}$$

As,$t\to \infty$, we get $0\le N\le \frac{\Delta }{\mu }$, the theory of differential equation^27^ in the region.

${\rm Z}_{k} = \{\left( S,{I}_{k},{I}_{kd}\right)\epsilon {R}^{3}+ : N\le \frac{\Delta }{\mu } \}$ For the autonomous system representing the Kidney disease-only model, given by (7), any solution that starts in ${Z}_{k}$ will stay within ${Z}_{k}$ for all $t\ge 0$

#### Kidney disease sub-model with disease-free equilibrium (DFE)

By equating Eq. (10) to zero $\frac{dS}{dt}=\frac{{dI}_{k}}{dt}=\frac{d{I}_{kd}}{dt}=0$

The disease-free equilibrium (DFE) of the COVID-19-only model system (7) is obtained by setting each of the systems of model system (10) to zero. Also, at the DFE, there are no infections. Thus, the DFE of the COVID-19-only model (10) is given by ${\Omega }_{0k}=\left( S,{I}_{k},{I}_{kd}\right)=(\frac{\Delta }{\mu },\mathrm{0,0})$

#### Basic reproduction number ${R}_{0k}$

Employing the next-generation matrix method outlined in (Yang 2014), we derive the related next-generation matrix as

$$\begin{gathered} F = \left[ {\begin{array}{*{20}c} {\frac{{\phi_{2} \left( {I_{k} + \theta I_{kd} } \right)}}{N}S} \\ 0 \\ \end{array} } \right] \hfill \\ V = \left[ {\begin{array}{*{20}c} {\left( {\sigma_{1} + \mu } \right)I_{k} } \\ { - \sigma_{1} I_{k} + \mu I_{kd} } \\ \end{array} } \right] \hfill \\ \end{gathered}$$

Consequently, the terms for new infections, F and the subsequent transfer components, V are provided as follows:

$$\begin{gathered} F = \left[ {\begin{array}{*{20}c} {\phi_{2} } & {\phi_{2} \theta } \\ 0 & 0 \\ \end{array} } \right] \hfill \\ V = \left[ {\begin{array}{*{20}c} {\left( {\sigma_{1} + \mu } \right)} & 0 \\ { - \sigma_{1} } & \mu \\ \end{array} } \right] \hfill \\ {\text{So}},\,\,V^{ - 1} = \frac{1}{{\left( {\sigma_{1} + \mu } \right)\mu }}\left[ {\begin{array}{*{20}c} \mu & 0 \\ {\sigma_{1} } & {\left( {\sigma_{1} + \mu } \right)} \\ \end{array} } \right] \hfill \\ \end{gathered}$$

The next-generation matrix $F{V}^{-1}$’s leading eigenvalue, which is also known as the spectral radius, represents the fundamental reproductive number and is defined as:

9 $${R}_{ok}=\frac{{\phi }_{2}(\mu +\theta {\sigma }_{1})}{\left({\sigma }_{1}+\mu \right)\mu }$$

${R}_{ok}$ represents the anticipated count of secondary infections produced by a single infected person throughout their entire infectious phase within a wholly susceptible community.

##### Theorem 8

The DFE is locally asymptotically stable if ${R}_{Ok}< 1$ and unstable if ${R}_{Ok}>1$

We use the Jacobian matrix to ascertain the local stability of equilibrium points. For sub-model (7), the Jacobian matrix is given as

$$J=\left(\begin{array}{c}\frac{\partial {f}_{1}}{\partial S} \frac{\partial {f}_{1}}{\partial {I}_{k}} \frac{\partial {f}_{1}}{{I}_{kd}}\\ \frac{\partial {f}_{2}}{\partial S} \frac{\partial {f}_{2}}{\partial {I}_{k}} \frac{\partial {f}_{2}}{{I}_{kd}} \\ \frac{\partial {f}_{3}}{\partial S} \frac{\partial {f}_{3}}{\partial {I}_{k}} \frac{\partial {f}_{3}}{{I}_{kd} }\end{array}\right)$$

$$J = \left( {\begin{array}{*{20}c} { - \frac{{\emptyset_{2} \left( {I_{k} + \theta I_{kd} } \right)}}{N} - \mu - \frac{{\emptyset_{2} S}}{N} - \frac{{\emptyset_{2} \theta S}}{N}} \\ { \frac{{\emptyset_{2} \left( {I_{k} + \theta I_{kd} } \right)}}{N} \frac{{\emptyset_{2} S}}{N} {-}\sigma_{1} - \mu \frac{{\emptyset_{2} \theta S}}{N} } \\ { 0 \,\,\,\,\sigma_{1} - \mu } \\ \end{array} } \right)$$

At the disease-free equilibrium point ${\Omega }_{0k} ,$ the Jacobian matrix of the sub-model is given

$$J({\Omega }_{0k})=\left(\begin{array}{c}-\mu -\frac{{\varnothing }_{2}\Delta }{N\mu } -\frac{{\varnothing }_{2}\theta\Delta }{N\mu }\\ 0 \frac{{\varnothing }_{2}\Delta }{N\mu }-\left({\sigma }_{1}+\mu \right) \frac{{\varnothing }_{2}\theta\Delta }{N\mu }\\ 0 {\sigma }_{1} -\mu \end{array}\right)$$

For $J({\Omega }_{0k})$ the eigenvalues is λ =  − μ, and the other eigenvalues can be swiftly obtained using the submatrix

$${J}_{2}=\left(\begin{array}{cc}{\phi }_{2}-\left({\sigma }_{1}+\mu \right)& {\upphi }_{2}\theta \\ {\sigma }_{1}& -\mu \end{array}\right)$$

We must show that ${J}_{2}^{\prime}s$ trace is negative, and its determinant is positive to determine the local stability of the disease-free equilibrium point.

Trc $\left({J}_{2}\right)={\phi }_{2}-({\tau }_{2}+2\mu ),$ which is less than zero. $if {\phi }_{2}<({\tau }_{2}+2\mu )$

det $$\left({J}_{2}\right)=-{\phi }_{2}\left(\mu +\theta {\sigma }_{1}\right)+\mu (\mu +{\sigma }_{1})$$

This value is greater than zero if $\frac{{\phi }_{2}\left({\theta \sigma }_{1}+\mu \right)}{\mu (\mu +{\sigma }_{1})}<1$ that is ${\text{det}}\left({J}_{2}\right)>0$ if ${R}_{0k}<1$ and ${\text{det}}\left({J}_{2}\right)<0$ if ${R}_{0k}>1$

For the kidney disease sub-model, the disease-free equilibrium point is stable when ${R}_{ok} < 1$ and unstable when ${R}_{ok} > 1$.

##### Theorem 9

Only when ${R}_{ok}>1$ does the endemic equilibrium point exist?

By resolving the above system of equations, we were also able to determine the endemic (disease present) equilibrium point of the renal disease sub-model:

$$\begin{gathered} \Delta - f_{k} S - \mu S = 0 \hfill \\ f_{k} S - \sigma_{1} I_{k} - \mu I_{k} = 0 \hfill \\ \sigma_{1} I_{k} - \mu I_{kd} = 0 \hfill \\ \end{gathered}$$

Here $${f}_{k}=\frac{{\phi }_{2}[{I}_{k}+\theta {{\text{I}}}_{{\text{kd}}}]}{N}$$

Solving the equation

${S}^{*}=\frac{\Delta }{{f}_{k}^{*}+\mu }$, ${I}_{k}^{*}=\frac{{f}_{k}^{*}{S}^{*}}{\mu +{\sigma }_{1}},{I}_{kd}^{*}=\frac{{\sigma }_{1}{f}_{k}^{*}{S}^{*}}{\mu (\mu +{\sigma }_{1})}$ applying this value we get,

$$\begin{gathered} f_{k}^{*} = \frac{{\phi_{2} \left[ {I_{k}^{*} + \theta {\text{I}}_{{{\text{kd}}}}^{*} } \right]}}{N} = \frac{{\phi_{2} f_{k}^{*} S^{*} }}{N}\left( {\frac{1}{{\mu + \sigma_{1} }} + \frac{{\theta \sigma_{1} }}{{\mu \left( {\mu + \sigma_{1} } \right)}}} \right) \hfill \\ f_{k}^{*} = \frac{{\mu \phi_{2} }}{{\left( {\mu + \sigma_{1} } \right)}}\left[ {1 + \frac{{\theta \sigma_{1} }}{\mu }} \right] - \mu \hfill \\ f_{k}^{*} = \mu \frac{{\phi_{2} \left( {\mu + \theta \sigma_{1} } \right)}}{{\mu \left( {\mu + \sigma_{1} } \right)}} - \mu \hfill \\ f_{k}^{*} = \mu \left( {R_{0k} - 1} \right) \hfill \\ \end{gathered}$$

Hence the endemic equilibrium point exists when ${R}_{ok} > 1$

#### Global stability of DFE

##### Theorem 10

The disease-free equilibrium point of the Kidney disease-sub model (7) is globally asymptotically stable. If ${R}_{ok}<1$

*Proof* Considering the Lyapunov function

10 $$T=\mu {I}_{k}+{\phi }_{2}\theta {I}_{kd}$$

Differentiating with respect to time

$$\begin{gathered} \frac{dT}{{dt}} = \mu \frac{{dI_{k} }}{dt} + \phi_{2} \theta \frac{{dI_{kd} }}{dt} \hfill \\ = \mu \phi_{2} \left( {\frac{{I_{k} + \theta I_{kd} }}{N}} \right)S - \mu \left( {\sigma_{1} + \mu } \right)I_{k} + \phi_{2} \theta \sigma_{1} I_{k} - \phi_{2} \theta \mu I_{kd} \hfill \\ \frac{dT}{{dt}} \le \mu \phi_{2} \left( {I_{k} + \theta I_{kd} } \right) - \mu \left( {\sigma_{1} + \mu } \right)I_{k} + \phi_{2} \theta \sigma_{1} I_{k} - \phi_{2} \theta \mu I_{kd} \hfill \\ \le \mu \phi_{2} I_{k} - \mu \left( {\sigma_{1} + \mu } \right)I_{k} + \phi_{2} \theta \sigma_{1} I_{k} \hfill \\ \le \phi_{2} (\theta \sigma_{1} + \mu )I_{k} - \mu \left( {\sigma_{1} + \mu } \right)I_{k} \hfill \\ \le R_{ok} \mu \left( {\sigma_{1} + \mu } \right)I_{k} {-}\mu \left( {\sigma_{1} + \mu } \right)I_{k} \hfill \\ \le (R_{ok} - 1)\mu \left( {\sigma_{1} + \mu } \right)I_{k} \hfill \\ \le 0,\,\,{\text{for}}\,\,R_{ok} \le 1 \hfill \\ \end{gathered}$$

since all the model parameters are positive, so that $\frac{dT}{dt}\le 0$ for ${R}_{ok}\le 1$, with $\frac{dT}{dt}=0$ when ${I}_{k}={I}_{kd}=0$. Using $\left({I}_{k},{I}_{kd}\right)=(\mathrm{0,0})$ into the Kidney disease only sub- model (7) represents that $S\to \frac{\Delta }{\mu }$ as $t\to \infty$. Hence $T$ is a Lyapunov function on ${\Omega }_{0k}$ and the largest compact invariant set in $\{\left( S,{I}_{k},{I}_{kd}\right)\in {\Omega }_{k}:\frac{dT}{dt}=0\}$ is ${\Omega }_{0k}$. So every solution of (7), with an initial condition in ${\Omega }_{k}$ approaches ${\Omega }_{0k}$, as $t\to \infty$ whenever ${R}_{ok}\le 1$.

##### Theorem 11

In the kidney disease-only model, the equilibrium point indicating the existence of the disease is globally stable when ${R}_{0k}$ $> 1$.

Denote the endemic equilibrium is denoted by ${E}_{k}=({S}^{*},{I}_{k}^{*},{I}_{kd}^{*}$), At the steady state, the force of infection ${f}_{k}$ is represented as:

11 $${f}_{k}^{*}=\frac{{\phi }_{2}({I}_{k}^{*}+\theta {I}_{kd}^{*})}{{S}^{*}+{I}_{k}^{*}+{I}_{kd}^{*}}$$

In the sub-model (7), we obtain by setting the right-hand sides equal to zero

$${S}^{*}=\frac{\Delta }{({f}_{k}^{*}+\mu )}$$

$${I}_{k}^{*}=\frac{\Delta {f}_{k}^{*}}{\left({f}_{k}^{*}+\mu \right)\mu }$$

$${I}_{kd}^{*}=\frac{\Delta {\sigma }_{1}{f}_{k}^{*}}{\left({f}_{k}^{*}+\mu \right)\mu }$$

Using (10),

12 $$\left(\mu +{\sigma }_{1}\right){f}_{k}^{*}+\mu \left(\mu +{\sigma }_{1}\right)-{\phi }_{2}\left({\theta \sigma }_{1}+\mu \right)=0$$

The linear Eq. (12) has a unique positive solution given by

$${f}_{k}^{*}=\frac{{\phi }_{2}\left({\theta \sigma }_{1}+\mu \right)-\mu \left(\mu +{\sigma }_{1}\right)}{(\mu +{\sigma }_{1})}$$

$$\left(\mu +{\sigma }_{1}\right){f}_{k}^{*}=\mu \left(\mu +{\sigma }_{1}\right)({R}_{ok}-1)$$

$${f}_{k}^{*}=\mu ({R}_{ok}-1)$$

This has biological significance when ${R}_{ok}>1$. It is mentioned that ${R}_{ok}<1$ implies that ${\phi }_{2}\left(\theta {\sigma }_{1}+\mu \right)-\mu \left(\mu +{\sigma }_{1}\right)<0.$ When this occurs, the force of infection ${f}_{k}$ is negative, suggesting that the disease’s equilibrium point shifts to global stability.

#### Analysis of sensitivity for the kidney disease model

Equation (7) specifies the renal sub-model and the examination of sensitivity for its basic reproduction number uses Yang’s (2014) tabilize forward sensitivity index for that basic reproduction number.

$${R}_{ok}=\frac{{\phi }_{2}(\mu +\theta {\sigma }_{1})}{\mu \left(\mu +{\sigma }_{1}\right)}$$

$${S}_{{\phi }_{2}}=\frac{\partial {R}_{0k}}{\partial {\varnothing }_{2}} \frac{{\phi }_{2}}{{R}_{0c}}=\frac{(\mu +\theta {\sigma }_{1})}{\mu \left(\mu +{\sigma }_{1}\right)} \frac{{\phi }_{2}}{\frac{{\phi }_{2}(\mu +\theta {\sigma }_{1})}{\mu \left(\mu +{\sigma }_{1}\right)}}=+1$$

$${S}_{{\sigma }_{1} }=\frac{\partial {R}_{0k}}{\partial {\upsigma }_{1}} \frac{{\sigma }_{1}}{{R}_{0k}}=\frac{{\phi }_{2}(\theta -1)}{{\left({\sigma }_{1}+\mu \right)}^{2}} \frac{{\sigma }_{1}}{\frac{{\phi }_{2}(\mu +\theta {\sigma }_{1})}{\mu \left(\mu +{\sigma }_{1}\right)}}=\frac{{\sigma }_{1}\mu (\theta -1)}{\left({\sigma }_{1}+\mu \right)(\mu +\theta {\sigma }_{1})}$$

$${S}_{\uptheta }=\frac{\partial {R}_{0k}}{\partial\uptheta } \frac{\uptheta }{{R}_{0k}}=\frac{{{\phi }_{2}\sigma }_{1}}{\mu \left(\mu +{\sigma }_{1}\right)}\frac{\uptheta }{\frac{{\phi }_{2}(\mu +\theta {\sigma }_{1})}{\mu \left(\mu +{\sigma }_{1}\right)}}=\frac{\uptheta {\upsigma }_{1}}{(\mu +\theta {\sigma }_{1})}$$

$${S}_{\upmu }=\frac{\partial {R}_{0k}}{\partial\upmu } \frac{\upmu }{{R}_{0k}}=-\frac{{\upphi }_{2}\left({\mu }^{2}+2\mu {\sigma }_{1}+\theta {\sigma }_{1}^{2}\right)}{{\mu }^{2}{\left(\mu +{\sigma }_{1}\right)}^{2}}\frac{\upmu }{\frac{{\phi }_{2}\left(\mu +\theta {\sigma }_{1}\right)}{\mu \left(\mu +{\sigma }_{1}\right)}}=-\frac{\left({\mu }^{2}+2\mu {\sigma }_{1}+\theta {\sigma }_{1}^{2}\right)}{(\mu +\theta {\sigma }_{1})}$$

Based on the sensitivity indices presented in Table 2, several observations can be made regarding the factors influencing the spread of kidney disease: 1. The contact rate specific to kidney disease is represented by ${\phi }_{2}$ exhibit a pronounced positive correlation with the disease’s propagation. This implies that as these rates increase, the disease spreads more aggressively. 2. The parameter adjusting for the enhanced transmission of kidney disease among co-infected individuals and those in the end-stage of the disease, denoted as $\theta$, and Progression rates ${\sigma }_{1}$ also positively influences the spread of the disease. This suggests a higher transfer rate among the co-infected exacerbates the spread of the disease. 3. Conversely, certain parameters, namely μ, mitigate the spread of kidney disease. Specifically, elevating the values of this parameter leads to a reduction in the number of individuals afflicted with kidney disease.

**Table 2:** Sensitivity indices for the kidney disease-only sub-model.

Parameter	Description	Sensitivity indices
${\sigma }_{1}$	Progression rates to fully increased kidney disease by Compartments ${I}_{k}$	$+0.036$
${\phi }_{2}$	Contact rate of kidney disease	+ 1
$\mu$	Natural death rate	$-0.151$
$\theta$	The parameter adjusting for the enhanced transmission of kidney disease among co-infected individuals and those in the end-stage of the disease	$+0.9974$

#### COVID-19 and kidney disease full model

By analyzing the equations’ right-hand sides, we could derive the equilibrium locations for the entire model (1).

13 $$\begin{gathered} \Delta - f_{k} S - {\text{f}}_{{\text{c}}} S - \mu S = 0 \hfill \\ f_{k} S - \sigma_{1} I_{k} - \alpha_{1} f_{c} I_{k} + {\text{f}}_{{\text{k}}} R + \tau_{2} I_{kc} - \mu I_{k} = 0 \hfill \\ \phi R + f_{c} S - {\text{f}}_{{\text{k}}} I_{c} - \tau_{1} I_{c} - \mu I_{c} = 0 \hfill \\ \alpha_{1} f_{c} I_{k} + {\text{f}}_{{\text{k}}} I_{c} - \sigma_{2} I_{kc} - \tau_{2} I_{kc} - \mu I_{kc} = 0 \hfill \\ \sigma_{1} I_{k} + \tau_{3} I_{kdc} - \alpha_{2} f_{c} I_{kd} - \mu I_{kd} = 0 \hfill \\ \sigma_{2} I_{kc} + \alpha_{2} f_{c} I_{kd} - \tau_{3} I_{kdc} - \mu I_{kdc} = 0 \hfill \\ \tau_{1} I_{c} - f_{k} R - \phi R - \mu R = 0 \hfill \\ \end{gathered}$$

where the forces of infection ${f}_{k}$ and ${f}_{c}$ are identical to those in Eqs. (5) and (10). The whole model’s disease-free equilibrium point $({\Omega }_{0ck})$ is then calculated as

14 $${\Omega }_{0ck} =(\frac{\Delta }{\mu },\mathrm{0,0},\mathrm{0,0},\mathrm{0,0})$$

We have now calculated the basic reproduction number ${R}_{0}$ of the complete model using the next-generation matrix. Using the notation of the diseased states $({I}_{c} , {I}_{k} ,{I}_{kd}, {I}_{kc}, {I}_{kdc} )$, Given the vector differential equations form $\frac{dX}{dt}=F\left(x\right)-V(x)$, where $V(x) = {V}^{-}(x)-{V}^{+}(x). F (x)$ is the rate at which new infections arise in compartments, ${V}^{+} (x)$ is the rate at which people are transferred into the compartment, and ${V}^{-} \left(x\right)$ is the rate at which people are transferred out of the compartments ${I}_{k},{I}_{c},{I}_{kc},{I}_{kd},{I}_{kdc}$

$$F\left(x\right)=\left(\begin{array}{c}{f}_{k}S+{{\text{f}}}_{{\text{k}}}R\\ {f}_{c}S\\ {{\text{f}}}_{{\text{k}}}{I}_{c}\\ 0\\ 0\end{array}\right)and V(x)=\left(\begin{array}{c}{(\sigma }_{1}+{\alpha }_{1}{f}_{c}+\mu ){I}_{k}-{\tau }_{2}{I}_{kc}\\ ({{\text{f}}}_{{\text{k}}}+{\tau }_{1}+\mu {)I}_{c}\\ ({\sigma }_{2}+{\tau }_{2}+\mu ){I}_{kc}{-\alpha }_{1}{f}_{c}{I}_{k}\\ \left({\alpha }_{2}{f}_{c}+\mu \right){I}_{kd}-{\sigma }_{1}{I}_{k}-{\tau }_{3}{I}_{kdc}\\ ({\tau }_{3}+\mu {)I}_{kdc}-{\sigma }_{2}{I}_{kc}-{\alpha }_{2}{f}_{c} {I}_{kd}\end{array}\right)$$

At$$,{E}_{0}$$

$$F=\left(\begin{array}{ccccc}{\phi }_{2} & 0& \theta & \theta & \theta \\ 0& {\phi }_{1}& \gamma & 0& \gamma \\ 0& 0& 0& 0& 0\\ 0& 0& 0& 0& 0\\ 0& 0& 0& 0& 0\end{array}\right)$$

$$V=\left(\begin{array}{ccccc}{(\sigma }_{1}+\mu )& 0& -{\tau }_{2}& 0& 0\\ 0& {\tau }_{1}+\mu & 0& 0& 0\\ 0& 0& ({\sigma }_{2}+{\tau }_{2}+\mu )& 0& 0\\ -{\sigma }_{1}& 0& 0& \mu & -{\tau }_{3}\\ 0& 0& -{\sigma }_{2}& 0& ({\tau }_{3}+\mu )\end{array}\right)$$

$${V}^{-1}=\left(\begin{array}{ccccc}\frac{1}{{(\sigma }_{1}+\mu )}& 0& -{\tau }_{2}& 0& 0\\ 0& \frac{1}{{\tau }_{1}+\mu }& 0& 0& 0\\ 0& 0& \frac{1}{{\sigma }_{2}+{\tau }_{2}+\mu }& 0& 0\\ -\frac{{\sigma }_{1}}{{(\sigma }_{1}+\mu )}& 0& 0& \frac{1}{\mu }& -{\tau }_{3}\\ 0& 0& -\frac{{\sigma }_{2}}{({\sigma }_{2}+{\tau }_{2}+\mu ){(\tau }_{3}+\mu )}& 0& \frac{1}{{\tau }_{3}+\mu }\end{array}\right)$$

$${FV}^{-1}=\left(\begin{array}{ccccc}\frac{{\phi }_{2}(\theta {\sigma }_{1}+\mu )}{\mu {(\sigma }_{1}+\mu )} & 0& \frac{\theta }{\left({\sigma }_{2}+{\tau }_{2}+\mu \right)}& \frac{\theta \Delta }{\mu }& \frac{\theta \Delta }{\mu }\\ 0& \frac{{\phi }_{1}\Delta }{\mu ({\tau }_{1}+\mu )}& \gamma \frac{\Delta }{\mu ({\sigma }_{2}+{\tau }_{2}+\mu )}& 0& \gamma \frac{\Delta }{\mu }\\ 0& 0& 0& 0& 0\\ 0& 0& 0& 0& 0\\ 0& 0& 0& 0& 0\end{array}\right)$$

To determine the basic reproduction number ${R}_{ck}$ of the system, the eigenvalues can be employed, specifically by examining the spectral radius of the matrix ${FV}^{-1}.$ The eigenvalues can be determined by assessing the equation:

$$\begin{gathered} \det \left[ {FV^{ - 1} - \lambda I} \right] = 0 \hfill \\ |FV^{ - 1} - \lambda I| = \left| {\begin{array}{*{20}c} {\frac{{\phi_{2} \left( {\theta \sigma_{1} + \mu } \right)}}{{\mu (\sigma_{1} + \mu )}} - \lambda } & 0 & {\frac{\theta }{{\left( {\sigma_{2} + \tau_{2} + \mu } \right)}}} & \theta & \theta \\ 0 & {\frac{{\phi_{1} }}{{\left( {\tau_{1} + \mu } \right)}} - \lambda } & {\frac{\gamma }{{\left( {\sigma_{2} + \tau_{2} + \mu } \right)}}} & 0 & \gamma \\ 0 & 0 & { - \lambda } & 0 & 0 \\ 0 & 0 & 0 & { - \lambda } & 0 \\ 0 & 0 & 0 & 0 & { - \lambda } \\ \end{array} } \right| = 0 \hfill \\ \end{gathered}$$

Here eigenvalues are ${\lambda }_{1}=\frac{{\phi }_{2}(\theta {\sigma }_{1}+\mu )}{\mu {(\sigma }_{1}+\mu )},{\lambda }_{2}=\frac{{\phi }_{1}}{\left({\tau }_{1}+\mu \right)},{\lambda }_{3}=0,{\lambda }_{4}=0,{\lambda }_{5}=0$

Thus, it can be concluded that the COVID-19 and kidney disease co-infection model has a reproduction number given by ${R}_{ck}=\{{R}_{oc},{R}_{ok}\}$;

where $${R}_{0k}=\frac{{\phi }_{2}(\theta {\sigma }_{1}+\mu )}{\mu {(\sigma }_{1}+\mu )}$$ and $${R}_{0c}=\frac{{\phi }_{1}}{\left({\tau }_{1}+\mu \right)}$$

#### Stability of ${\Omega }_{0ck}$ for the full co-infection model

##### Theorem 12

When ${R}_{ck}>1$, model (1) has ${(\Omega }_{0ck})$ that is locally asymptotically stable.

The eigenvalues of each equilibrium were used to examine its local stability (Fudolig and Howard, 2020). The eigenvalues are found in the Jacobian matrix, which each equilibrium has replaced. The model (1)’s Jacobian matrix can be described as

$$\Delta -{f}_{k}S-{{\text{f}}}_{{\text{c}}}S-\mu S=0$$

$${f}_{k}S-{\sigma }_{1}{I}_{k}-{\alpha }_{1}{f}_{c}{I}_{k}+{{\text{f}}}_{{\text{k}}}R+{\tau }_{2}{I}_{kc}-\mu {I}_{k}=0$$

$${f}_{c}S-{{\text{f}}}_{{\text{k}}}{I}_{c}-{\tau }_{1}{I}_{c}-\mu {I}_{c}=0$$

$${\alpha }_{1}{f}_{c}{I}_{k}+{{\text{f}}}_{{\text{k}}}{I}_{c}-{\sigma }_{2}{I}_{kc}-{\tau }_{2}{I}_{kc}-\mu {I}_{kc}=0$$

$${\sigma }_{1}{I}_{k}+{\tau }_{3}{I}_{kdc}-{\alpha }_{2}{f}_{c} {I}_{kd}-\mu {I}_{kd}=0$$

$${\sigma }_{2}{I}_{kc}+{\alpha }_{2}{f}_{c} {I}_{kd}-{\tau }_{3}{I}_{kdc}-\mu {I}_{kdc}=0$$

$${\tau }_{1}{I}_{c}-{f}_{k}R-\mu R=0$$

$$J=\left(\begin{array}{ccccccc}-\mu & -{\phi }_{2}& {-\phi }_{1}& -({\phi }_{2}\theta +{\phi }_{1}\gamma )& -{\phi }_{2}\theta & -({\phi }_{2}\theta +{\phi }_{1}\gamma )& 0\\ 0& {\phi }_{2}-({\sigma }_{1}+\mu ) & 0& {{\phi }_{2}\theta +\tau }_{2}& {\phi }_{2}\theta & {\phi }_{2}\theta & 0\\ 0& 0& {\phi }_{1}-({\tau }_{1}+\mu )& {\phi }_{1}\gamma & 0& 0& 0\\ 0& 0& 0& -({\sigma }_{2}+{\tau }_{2}+\mu )& 0& 0& 0\\ 0& {\sigma }_{1}& 0& 0& -\mu & {\tau }_{3}& 0\\ 0& 0& 0& {\sigma }_{2}& 0& -{(\tau }_{3}+\mu )& 0\\ 0& 0& {\tau }_{1}& 0& 0& 0& -\mu \end{array}\right)$$

At the disease-free equilibrium, we obtained the following characteristic polynomial:

15 $${Q}_{0} (\lambda ) =({\lambda }_{1}+\mu )({\lambda }_{2}+\mu )({\lambda }_{3}+\mu )({\lambda }_{4}+{\sigma }_{2}+{\tau }_{2}+\mu )({\lambda }_{5}-{\phi }_{2}+{\sigma }_{1}+\mu )({\lambda }_{6}-{\phi }_{1}+{\tau }_{1}+\mu )({\lambda }_{7}+{\tau }_{3}+\mu )$$

We get $${\lambda }_{1}=-\mu ,{\lambda }_{2}=-\mu ,{\lambda }_{3}=-\mu ,{\lambda }_{4}=-({\sigma }_{2}+{\tau }_{2}+\mu )$$

And $${\lambda }_{5}=-{\phi }_{2}+{\sigma }_{1}+\mu <0$$ and $${\lambda }_{6}=-{\phi }_{1}+{\tau }_{1}+\mu <0$$

$${\phi }_{2}<{\sigma }_{1}+\mu$$, $${\phi }_{1}<{\tau }_{1}+\mu$$

$$\frac{\mu }{(\theta {\sigma }_{1}+\mu }\frac{{\phi }_{2}(\theta {\sigma }_{1}+\mu )}{\mu ({\sigma }_{1}+\mu )}<1$$ and $$\frac{{\phi }_{1}}{{\tau }_{1}+\mu }<1$$

$$\frac{\mu }{(\theta {\sigma }_{1}+\mu )}{R}_{0k} <1$$ and $$\frac{{\phi }_{1}}{{(\tau }_{1}+\mu )}<1$$

So, $${R}_{ok}<1$$ and $${R}_{oc}<1$$

So, the co-infection full model (1), ${ \Omega }_{0ck}$ reaches local asymptotic stability as a disease-free equilibrium point.

#### Global stability analysis of co-infection full model

From the full model $\frac{dX}{dt}=F(X,Z)$, $\frac{dZ}{dt}=T\left(X,Z\right), T\left(X,0\right)=0,$

Here $X=(S,R)$ and $Z=({I}_{k},{I}_{c},{I}_{kc},{I}_{kd},{I}_{kdc})$. In this case, representation $X,$ which belongs to ${R}^{2}$ signifies the compartments of healthy individuals, while $Z$, a part of ${R}^{5}$, stands for the infected population compartments. The disease-free equilibrium state is denoted by ${U}_{0}=({X}_{0},0)$, where ${X}_{0}=(\frac{\Delta }{\mu },0)$

The following assumptions $({H}_{1})$ and $({H}_{2})$ ensure that ${\Omega }_{0ck}$ for ${R}_{ck}$ is globally asymptotically stable. $({H}_{1})$ For $\frac{dX}{dt} = F(X, 0),$ the equilibrium point ${U}_{0}$ is globally stable;

($H_{2} ) G\left( {X,Z} \right) = AZ {-} T\left( {X,Z} \right), \hat{G}\left( {X,Z} \right) \ge 0$ for $(X,Z) \in \Omega$, The feasible area of the constructed model is denoted by $\Omega$, and A = ${D}_{Z} T({U}_{0},0)$ is a Metzler matrix. From our co-infection mathematical model Eq. (1), we have $$\frac{dX}{dt}=F\left(X,Z\right)=\left[\begin{array}{c}\Delta -{f}_{k}S-{{\text{f}}}_{{\text{c}}}S-\mu S\\ {\tau }_{1}{I}_{c}-{f}_{k}R-\mu R\end{array}\right]$$

So, $$T\left(X,0\right)=\left[\begin{array}{c}\Delta -\mu S\\ 0\end{array}\right]$$ and

$$\begin{gathered} \frac{dZ}{{dt}} = T\left( {X,Z} \right) = \left[ {\begin{array}{*{20}c} {f_{k} S - \sigma_{1} I_{k} - \alpha_{1} f_{c} I_{k} + f_{k} R + \tau_{2} I_{kc} - \mu I_{k} } \\ {f_{c} S - f_{k} I_{c} - \tau_{1} I_{c} - \mu I_{c} } \\ { \alpha_{1} f_{c} I_{k} + f_{k} I_{c} - \sigma_{2} I_{kc} - \tau_{2} I_{kc} - \mu I_{kc} } \\ { \sigma_{1} I_{k} + \tau_{3} I_{kdc} - \alpha_{2} f_{c} I_{kd} - \mu I_{kd} } \\ {\sigma_{2} I_{kc} + \alpha_{2} f_{c} I_{kd} - \tau_{3} I_{kdc} - \mu I_{kdc} } \\ \end{array} } \right] \hfill \\ \hat{T}\left( {X,Z} \right) = AZ - T\left( {X,Z} \right) \hfill \\ So,\,\,\hat{T}\left( {X,Z} \right) = \left[ {\begin{array}{*{20}c} {\hat{T}_{1} \left( {X,Z} \right)} \\ {\hat{T}_{2} \left( {X,Z} \right)} \\ {\hat{T}_{3} \left( {X,Z} \right)} \\ {\hat{T}_{4} \left( {X,Z} \right)} \\ {\widehat{{T_{5} }}\left( {X,Z} \right)} \\ \end{array} } \right] = \left[ {\begin{array}{*{20}c} { - f_{k} S - f_{k} R + \alpha_{1} f_{c} I_{k} } \\ { - f_{c} S + f_{k} I_{c} } \\ { {-}\alpha_{1} f_{c} I_{k} - f_{k} I_{c} } \\ {\alpha_{2} f_{c} I_{kd} } \\ { - \alpha_{2} f_{c} I_{kd} } \\ \end{array} } \right] \hfill \\ \end{gathered}$$

Thus,${-\alpha }_{1}{f}_{c}{I}_{k}-{{\text{f}}}_{{\text{k}}}{I}_{c}<0$ and $-{\alpha }_{2}{f}_{c} {I}_{kd}<0$. From this, condition ${H}_{2}$ is not met. Consequently,${U}_{0}$ and subsequently, the disease-free equilibrium point ${\Omega }_{ck}$, cannot achieve global asymptotic stability.

## Parameter estimation

We have derived the values of the model parameters using authentic data from Bangladesh, encompassing both kidney disease and cumulative COVID-19 infected cases. The COVID-19 dataset, from the initial reporting date of March 8, 2020, to September 8, 2020, was collated daily and sourced from^28^. Concurrently, the data for Kidney disease from 2020 to 2023 was compiled every month and can be accessed^29^. To calibrate the model and deduce the parameter values from the data, we employed a hybrid approach combining least squares and Bayesian methods. Additionally, a nonlinear curve-fitting technique was employed, using MATLAB’s ‘fminsearch’ function Certain parameters were inferred from existing literature. For instance, based on Worldometer’s data, Bangladesh’s average life expectancy in 2020 was 72.72 years (macrotrends,2024), and we considered a subset population of 16,580,000. This led to the calculation of the natural mortality rate per month as the inverse of life expectancy, resulting in a value of $\upmu =\frac{1}{72.72\times 365}=0.000038$. Furthermore, the recruitment rate was approximated by manipulating the ratio of $\frac{\nabla }{\upmu }$ to yield the initial population, resulting in $\nabla =630$ individuals per day. Due to limited data on co-infections, we estimated certain co-infection related parameters, while others were deduced from actual data. During the estimation process, the initial conditions of the state variables were set as delineated in Table 3.

**Table 3:** Description of variables and parameters in the model equation.

Parameter	Description	Value	Reference
$\Delta$	Recruitment rate of the human population	630	Calculated
${\phi }_{1}$	Contact rate of Covid-19	0.1175	Fitted
${\phi }_{2}$	Contact rate of kidney disease	0.3425	Fitted
$\theta$	The parameter adjusting for the enhanced transmission of kidney disease among co-infected individuals and those in the end-stage of the disease	1.1	^8^
$\gamma$	Parameter accounting for the amplified transmissibility of COVID-19 in co-infected persons	1.0	Estimated
${\sigma }_{1}$	Progression rates to fully increased kidney disease by compartments ${I}_{k}$	0.15	Fitted
${\sigma }_{2}$	Progression rates to fully increased kidney disease by compartment ${I}_{kc}$	0.15	Fitted
${\alpha }_{1},{\alpha }_{2}$	Parameters denote adjustments for the susceptibility of individuals with kidney disease to contracting COVID-19 infection	1.3	^12^
${\tau }_{1}$	COVID-19 single-infection recovery rate	0.067	Estimated
${\tau }_{2}$	COVID-19 recovery rate in the compartment ${I}_{kc}$	0.067	Estimated
${\tau }_{3}$	Recovery rate among co-infected in compartment ${I}_{kdc}$ for COVID-19	0.067	Estimated
$\mu$	Human natural death rate	0.000038	Calculated

**Figure 3:**
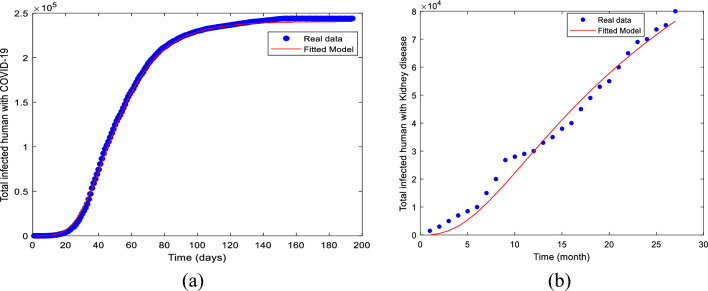
Model fitting with reported COVID-19 and kidney disease data.

Figure 3 illustrates the model’s fit for both cumulative COVID-19 infections and cumulative kidney infections. In Fig. 3a, the model’s output for COVID-19 infections is compared to the actual observed data of COVID-19 cases. Similarly, Fig. 3b demonstrates the alignment between the model’s simulation and the observed data for kidney disease infections. In both instances, solid lines represent the model’s simulated output, and dotted lines correspond to the actual observed data for the two diseases from Bangladesh. The comparison reveals a strong congruence between the model simulations and the actual data.

## Numerical simulations

To explore the co-infection dynamics between COVID-19 and Kidney disease in scenarios without treatment, we carried out numerical simulations using the combined COVID-19 and Kidney disease model. The majority of theoretical results from this investigation are illustrated through these simulations. For our computational study, we employed the ode45 function. Ode45, incorporated into MATLAB, is a non-stiff one-step solver based on the Runge–Kutta (4, 5) method. It stands out for its speed, accuracy, and stability. While it is superior to the Euler method in terms of efficiency, its true strength lies in its simplicity and stability, especially when juxtaposed with multi-step strategies. Despite consuming more computational time than other equivalent accuracy multi-step methods, the straightforward nature and user-friendliness of ode45 compensate for its computational demands. Parameters driving our simulations can be found in Table 3, along with the initial conditions set for the experiment $S=50000,{I}_{c}=500,{I}_{k}=300,{I}_{kd}=200,{I}_{kdc}=100,R=30$.

**Figure 4:**
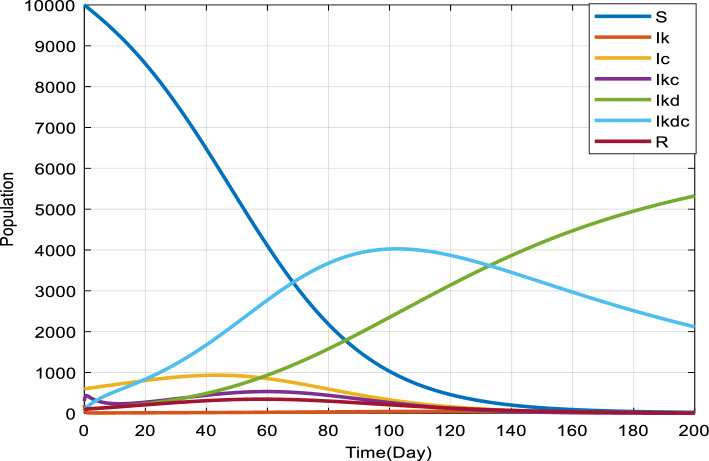
Solution of the comprehensive co-infection model using parameter values in Table 3.

Figure 4 showcases a series of time-dependent plots that illustrate the dynamics of the co-infection as it evolves over time. These plots have been constructed by numerically solving the co-infection model represented by Eq. (1). The solutions have been derived using the specific parameter values enumerated in Table 3. The progression depicted in each plot provides insights into the tabiliz of the diseases in the system and their interactions over the duration captured.

**Figure 5:**
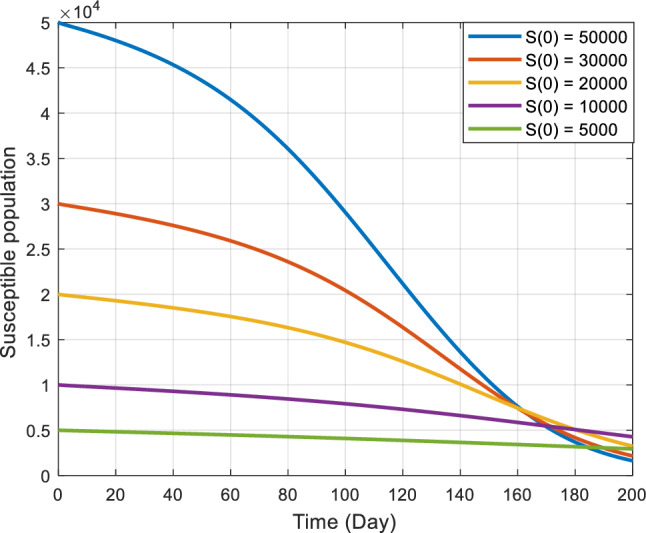
Graphical representation of the stability at the disease-free equilibrium point when ${R}_{ck}<1$ and ${R}_{ck}>1$.

**Figure 6:**
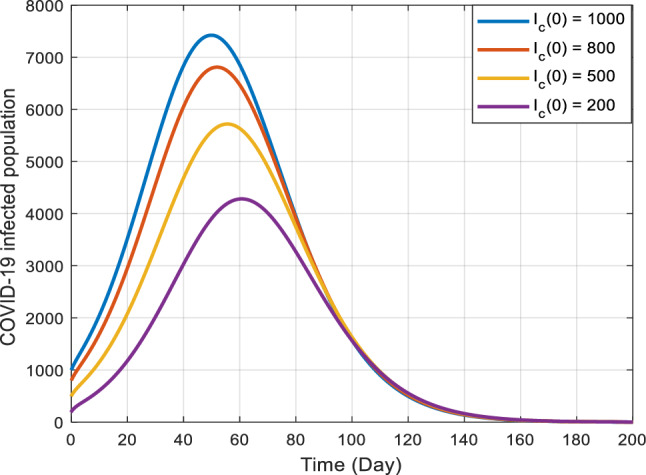
Graphical representation of the stability at the disease-free equilibrium point when ${R}_{0c}<1$ and ${R}_{0c}>1$.

**Figure 7:**
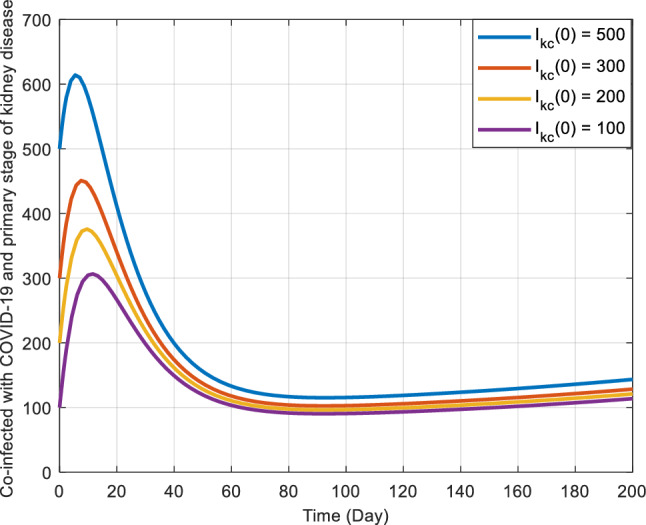
Graphical representation of the stability at the disease-free equilibrium point when ${R}_{ck}<1$ and ${R}_{ck}>1$.

Figures 5, 6 and 7 illustrate the stability characteristics solutions when subject to many initial circumstances. Specifically, Fig. 5 focuses on the initial conditions for the susceptible compartment within the COVID-19 sub-model. In contrast, Fig. 6 pertains to the infected compartment of the same sub-model. Lastly, Fig. 7 delves into the dynamics of those co-infected with COVID-19 and the primary stage of kidney disease. These figures provide valuable insights into how the system responds to changes in initial states, shedding light on the disease dynamics and potential interactions between the two health conditions.

**Figure 8:**
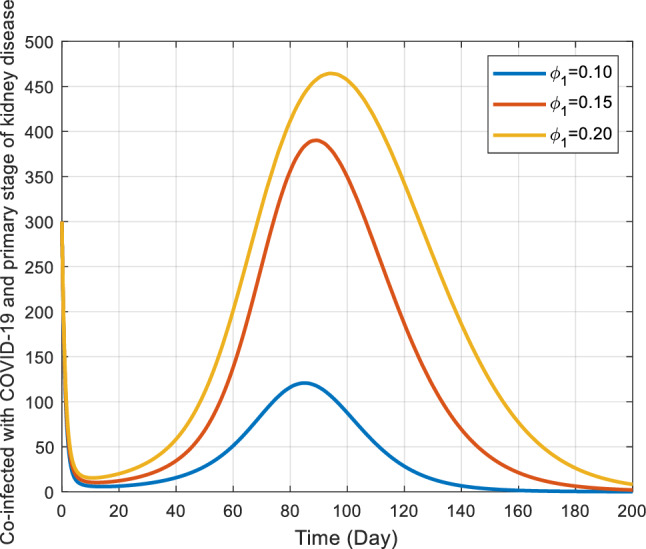
Behavior of ${I}_{kc}$ for the different values of ${\phi }_{1}$ and other values of the parameter in Table 3.

**Figure 9:**
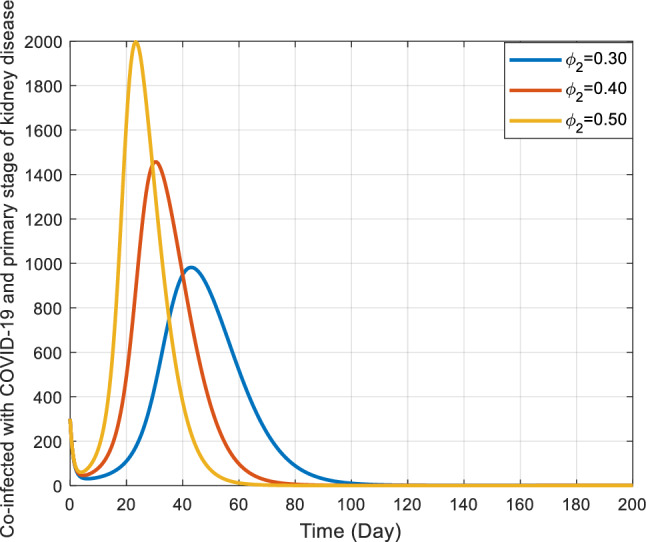
Behavior of ${I}_{kc}$ for the different values of ${\phi }_{2}$ and other parameter values in Table 3.

Figures 8 and 9 elucidate the influence of rates ${\phi }_{1}$ and ${\phi }_{2}$ on co-infected individuals within the ${I}_{kc}$ class. Notably, as these rates escalate, initially there’s a consequent increase in the count of individuals in the co-infected population, later after reaching a peak the count of individuals gradually declines. These rates, presumably, describe how quickly individuals leave or transition out of this co-infected population. The main observation drawn from Fig. 8 is that, as the ${\phi }_{1}$ increases, the rate of increase in the number of co-infected individuals also increases sharply and reaches a peak at almost the same time, declining gradually as the infected individuals recover or die. In context, the effect of ${\phi }_{2}$ on the number of infected individuals is very sensitive. As is noted in Fig. 9, for the largest value of ${\phi }_{2}$ the number of infected individuals quickly arrives at the peak. As ${\phi }_{2}$ decreases, it takes a relatively greater time for the number of co-infected individuals to reach at peak.

**Figure 10:**
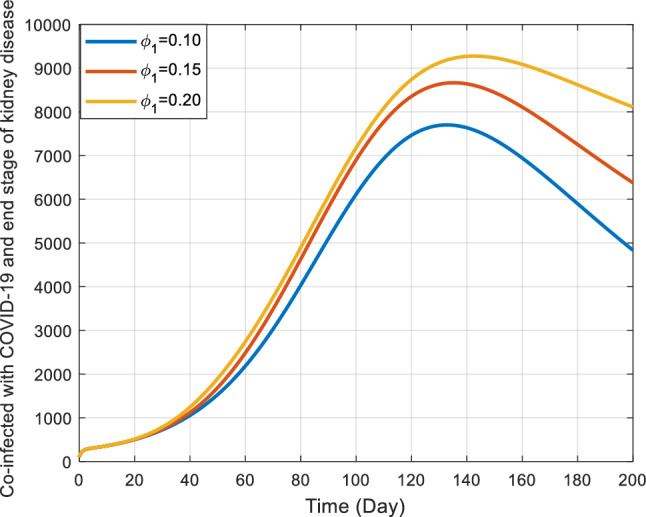
Behavior of ${I}_{kdc}$ for the different values of ${\phi }_{1}$ and other parameter values in Table 3.

**Figure 11:**
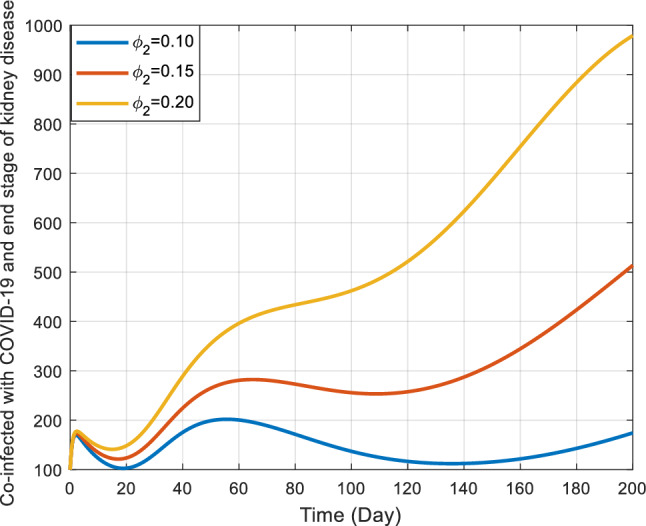
Effect of Contact rate of kidney disease interventions on co-infected populations.

Figures 10 and 11 offer detailed visual representations of how the rates ${\phi }_{1}$ and ${\phi }_{2}$ affect the co-infected individuals within the COVID-19 and end stage of kidney disease ${(I}_{kdc})$ class. These rates signify how individuals transition out of the co-infected state.

As ${\phi }_{1}$ increases, the co-infected individuals also increase for a certain period (around 130 days) and then decrease slowly. On the contrary, the dynamics of co-infected individuals in the end-stage kidney disease show some variation. For example, the largest value of ${\phi }_{2}$ there is an increasing trend in the number of co-infected individuals, so for moderate value of ${\phi }_{2}$. But for the lowest value of ${\phi }_{2}$ the trend of co-infected individuals shows up and down tabiliz. Interestingly, contrary to initial assumptions, the figures indicate that a rise in either ${\phi }_{1}$​ or ${\phi }_{2}$ corresponds to an uptick in the number of co-infected individuals within the ${I}_{kdc}$ class.

**Figure 12:**
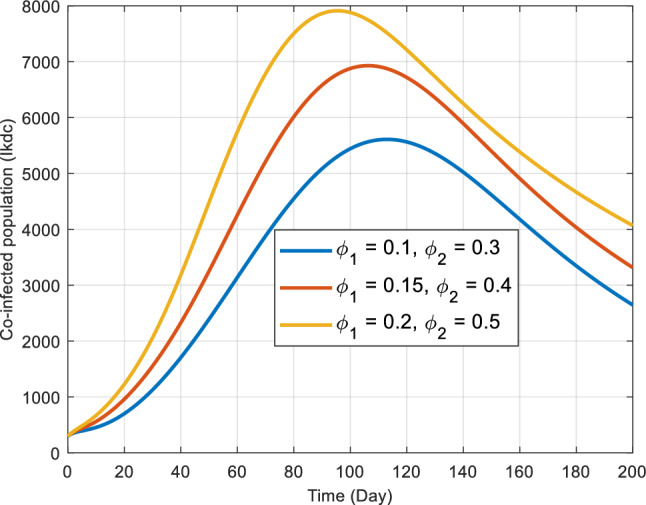
Impact of the contact rates $${\phi }_{1}$$ and $${\phi }_{2}$$ on the transmission dynamics of the co-infected ones ($${I}_{kdc})$$

In Fig. 12, we illustrate the influence of transfer rates to the co-infected class, stemming from each actively infected individual of the respective diseases. Specifically, this figure delves into the effects of contact rates about co-infected compartments of both COVID-19 and end-stage kidney disease (${I}_{kdc}$) as described in our system (1). The interactive effect of ${\phi }_{1}$ and ${\phi }_{2}$ depicts that the co-infected population rises to a peak at a particular time point and then decreases regardless of the different parameter combinations. However, the curves do not intersect for different levels of either ${\phi }_{1}$ or ${\phi }_{2}$ the trend of co-infected populations is similar for ${I}_{kdc}$ group.

**Figure 13:**
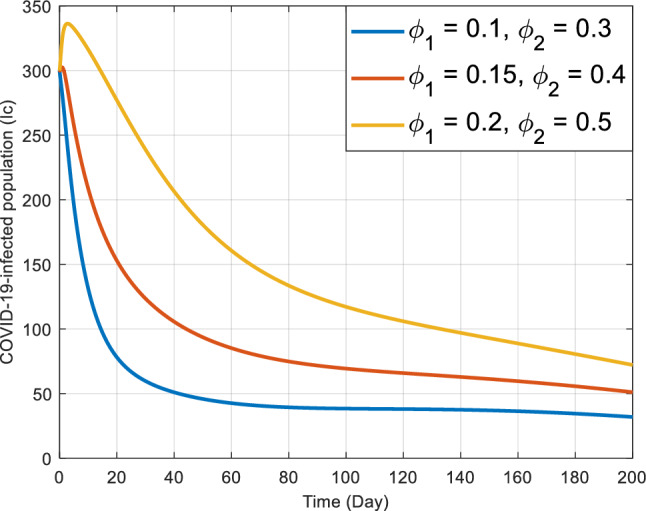
Impacts of the contact rates ${\phi }_{1}$ and ${\beta }_{2}$ on the dynamics of infected COVID-19 $({I}_{c} )$ transmission.

Figure 13 showcases the proliferation of co-infected as the effective contact rates vary. In contrast, the dynamics of individuals solely infected with COVID-19, to differing contact rates, are depicted in both Figs. 12 and 13. A notable observation is that the population in the state ${I}_{c}$ diminishes while in ${I}_{kdc}$ grows as transmission coefficients escalate. Crucially, these numerical observations echo our analytical insights drawn from the sensitivity analysis within the sub-models.

**Figure 14:**
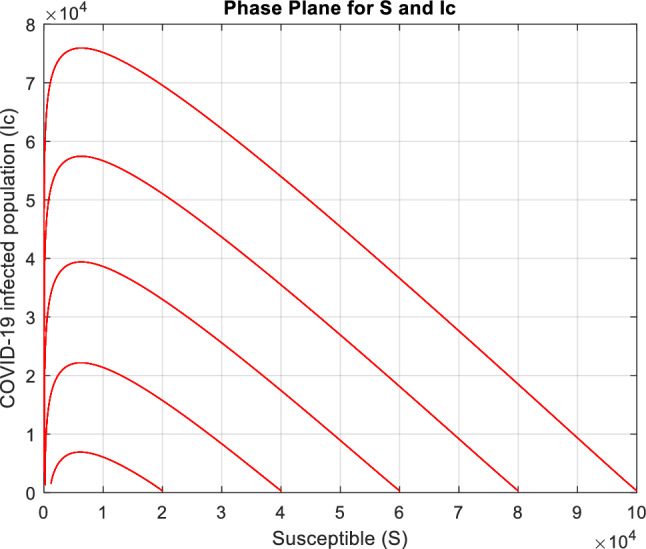
Phase plane illustrating the dynamical interplay between susceptible population individuals $S$ and infected COVID-19 individuals ${I}_{c}.$

Figure 14 shows an inverse relationship between the susceptible and COVID-19-infected populations. As the number of susceptible individuals rises, the number of those infected with COVID-19 decreases. This phase plane suggests that new infections decline as more individuals become less vulnerable or exposed to the virus (perhaps due to factors like vaccination, prior infection, or preventive measures).

**Figure 15:**
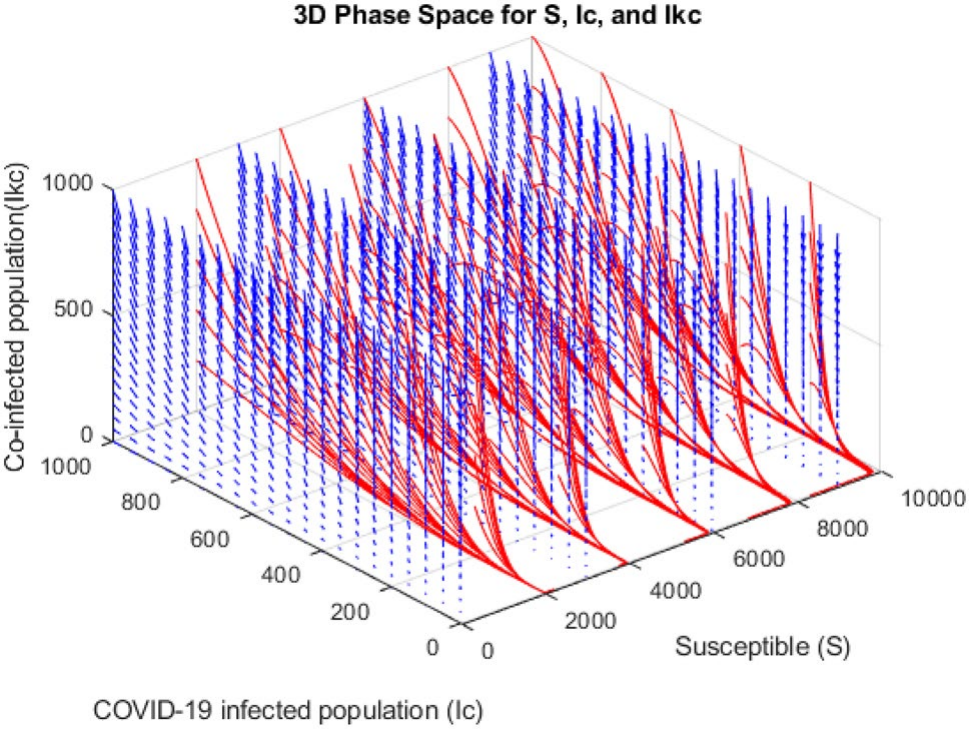
Phase portrait illustrating the dynamic interactions among the compartments $S,{I}_{c}$ and ${I}_{kc}$.

In Fig. 15, the graph reveals that as the number of infected solely with only COVID-19 grows, there is a corresponding increase in the population co-infected with both COVID-19 and the primary stage of kidney disease. Simultaneously, we observe a decline in the susceptible population. Intriguingly, when there’s a surge in the susceptible population, neither the co-infected nor the solely COVID-19-infected group shows a proportional rise. Instead, their numbers stabilise or remain consistent; they plateau or stay steady.

**Figure 16:**
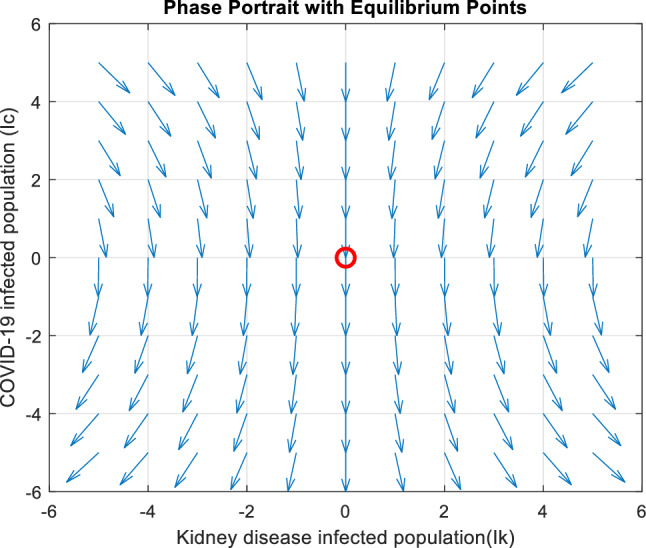
Phase portrait illustrating the dynamic interactions among the compartments ${I}_{k}$ and ${I}_{c}$.

In Fig. 16, the scatter plot shows a positive correlation between the number of kidney disease individuals and the number of COVID-19-infected people. Also, our analytical analysis shows that people with kidney disease are more likely to get COVID-19.

**Figure 17:**
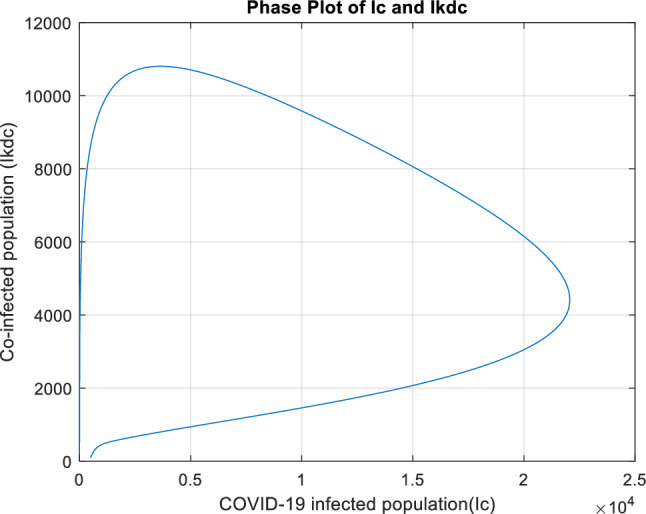
Phase plot illustrating the dynamic interactions among the compartments ${I}_{c}$ and ${I}_{kdc}$.

Figure 17 demonstrates a positive correlation between the two variables, suggesting that those infected with COVID-19 have a higher risk of being co-infected with COVID-19 and kidney disease. COVID-19 can damage the kidneys, leading to acute kidney injury and a sudden loss of kidney function. Acute kidney injury can be fatal and is more likely to occur in people with kidney disease.

## Conclusion

We developed a mathematical model to study the spread of co-infection between kidney disease and COVID-19. This model ensures solutions are positive and limited within a biologically meaningful range. We identified equilibrium points for the diseases separately and analysed their stability based on their basic reproduction numbers. We also examined the co-infection reproduction number and its sensitivity analysis, revealing that a rise in infection rates from either disease increases the co-infection risk. The key findings of the new development model are listed below;

- Our analysis found that if the infection rate for either COVID-19 or kidney disease increases, the risk of people getting both diseases increases significantly. This means that controlling the spread of each disease is crucial to reducing the overall risk of co-infections.
- Changing how easily each disease is transmitted (known as transmission coefficients) affects the diseases differently depending on their stage. For example, a transmission change might significantly impact someone who's just contracted the disease more than someone living with it for a while.
- We looked at how changes in the contact rate for COVID-19 $({\upphi }_{1})$ and the contact rate for kidney disease $({\upphi }_{2})$ affect the diseases. We found that these changes have different impacts depending on whether the kidney disease is in its early stage (primary) or late stage (end-stage). This means how each disease spreads and affects people can vary significantly based on the disease's progression.
- We identified specific points, called equilibrium points, for each disease. These points help us understand how likely the disease will remain in the population over time. If the number we calculate for these points is more than one, it suggests that the disease will continue to exist within the population. This is a key indicator for public health strategies, highlighting the need for ongoing disease management and control measures.

The observations drawn from the model are consistent with analytical conclusions from the sensitivity analysis, especially emphasising the critical role of reducing the susceptible population—potentially through measures like vaccination or natural immunity—to decrease new infections. The findings highlight the complex interplay of disease transmission and co-infections, presenting areas of concern and possible intervention points for effective disease control. The present results and models also maximise the benefits of simulation modelling to minimise the global health complexity of COVID-19 and kidney disease. The more effective strategies for reducing the impact of COVID-19 and kidney disease through optimal control methods are used in our forthcoming studies.

## Data Availability

The datasets used and/or analysed during the current study available from the corresponding author on reasonable request.
